# Antifungal Activity of *Eucalyptus* Oil against Rice Blast Fungi and the Possible Mechanism of Gene Expression Pattern

**DOI:** 10.3390/molecules21050621

**Published:** 2016-05-12

**Authors:** Li-Jun Zhou, Fu-Rong Li, Li-Jie Huang, Zhi-Rong Yang, Shu Yuan, Lin-Han Bai

**Affiliations:** 1 Key Laboratory of Bio-Resources and Eco-Environment of Ministry of Education, Animal Disease Prevention and Food Safety Key Laboratory of Sichuan Province, College of Life Sciences, Sichuan University, Chengdu 610064, China; zhouzhou124@126.com (L.-J.Z.); lmm201411@163.com (F.-R.L.); huanglijie1104@163.com (L.-J.H.); bioyang@163.com (Z.-R.Y.); 2 College of Life Sciences and College of Resources, Sichuan Agricultural University, Ya’an 625000, China

**Keywords:** *E. grandis* × *E. urophylla* oil, antifungal effects, *M. grisea*, mRNA Genome Array, biological control

## Abstract

*Eucalyptus* oil possesses a wide spectrum of biological activity, including anti-microbial, fungicidal, herbicidal, acaricidal and nematicidal properties. We studied anti-fungal activities of the leaf oil extracted from *Eucalyptus. grandis* × *E. urophylla*. Eleven plant pathogenic fungi were tested based on the mycelium growth rates with negative control. The results showed that *Eucalyptus* oil has broad-spectrum inhibitory effects toward these fungi. Remarkable morphological and structural alterations of hypha have been observed for *Magnaporthe grisea* after the treatment. The mRNA genome array of *M*. *grisea* was used to detect genes that were differentially expressed in the test strains treated by the *Eucalyptus* oil than the normal strains. The results showed 1919 genes were significantly affected, among which 1109 were down-regulated and 810 were up-regulated (*p* < 0.05, absolute fold change >2). According to gene ontology annotation analysis, these differentially expressed genes may cause abnormal structures and physiological function disorders, which may reduce the fungus growth. These results show the oil has potential for use in the biological control of plant disease as a green biopesticide.

## 1. Introduction

Agricultural studies have focused on the biocontrol of plant disease for a long time. Discovery of antifungal and germicidal compounds from plants is an efficient way to create new pollution-free pesticides. Many plants have antimicrobial activities that are related to their antimicrobial constituents, including alkaloids, terpenes, polysaccharide, esters, ketones, and quinones [1]. Effective components extracted from plants have promising potential for this purpose because of their high efficacy, low toxicity, and selective characteristics [2]. Various biopesticides have been developed that contain nicotine, rotenone, matrine, toosendanin and other similar compounds as their main component; for example, Green Gold, Econeem, Akign™, Neem Azal™, and Saferin [3].

Essential oils are basic active natural pesticides and disease control compounds found in a wide range of plants. Essential oils have become a major research focus in recent years for their effective disease and insect pest controlling functions. The essential oil of *Eucalyptus* possesses a wide spectrum of biological activity [4], including anti-microbial, fungicidal, insecticidal/insect repellent, herbicidal, acaricidal and nematicidal properties. The essential oil of *Eucalyptus* may be obtained from various different plant parts, however, the highest concentration of *Eucalyptus* essential oil has been found in the leaves. The dominant compounds in *Eucalyptus* oil include 1,8-cineole, limonene, *p*-cymene, γ-terpinene, α-pinene, α-terpineol, camphene, linalool, and ocimene [5,6,7,8,9,10,11]. The antimicrobial action of the essential oil of *Eucalyptus* is well known. Considerable research has indicated that the *Eucalyptus* extracts such as *E. citriodora*, *E. globulus* and *E. staigeriana*, have clear antimicrobial action against many kinds of pathogenic microbes [7,12,13,14,15]. However, few studies into the inhibitory mechanisms have been conducted. 

*Eucalyptus* agriculture has significant economic and ecological benefits. However, the leaves of *Eucalyptus* are often burned as fuel or eventually discarded, which have not been well used until now. In this study we analyzed EO, the essential oil of *E. grandis* × *E. urophylla* oil, a fast-growing hybrid clone between *E. grandis* and *E. urophylla*, which is an economically important pulp tree that is widely grown in many provinces in south China. This pulp tree (Guanglin 9 cultivar) was introduced to Yuping, a small town of Sichuan Province in 2008. The main constituents of local *Eucalyptus* oil are α-pinene (24.78%), 1,8-cineole (45.57%), terpinyl acetate (8.34%), α-terpineol (2.51%), isoborneol (1.45%), camphene (0.60%) and linalool (0.15%) [16]. Isoborneol, linalool, α-terpineol, 1,8-cineole, α-pinene and camphene have known antimicrobial properties [17,18,19,20,21,22,23]. Known antimicrobial components account for over 75.1% of the total volatile oil. In general, the strong antimicrobial activity is related not only to a high content of one major component such as 1,8-cineole, but also to the presence of moderate and minor compounds [12].

Here we report the findings of systematic research into the main components, the inhibitive activities, the morphological variations, and related molecular mechanisms to improve understanding of the broad-spectrum antifungal effect of the leaf oil of *E. grandis* × *E. urophylla*.

## 2. Results and Discussion

### 2.1. Inhibitory Activities of the Eucalyptus Oil on Plant Pathogenic Fungi

Table 1 shows the inhibitory activities of the *Eucalyptus* oil with a final concentration of 2.5 mg/mL on 11 plant pathogenic fungi based on the mycelium growth rates with negative control. It is clear that the *Eucalyptus* oil had broad-spectrum inhibitory effects to these fungi (Table 1). We observed a higher inhibition rate of serious plant pathogenic fungi like *S. turcica*, *M. grisea*, *B. cinerea*, *F. graminearum* and *B. maydis* relative to the control. Based on these results, we conclude that the oil has potential for use in the biological control of plant disease.

**Figure 1:**
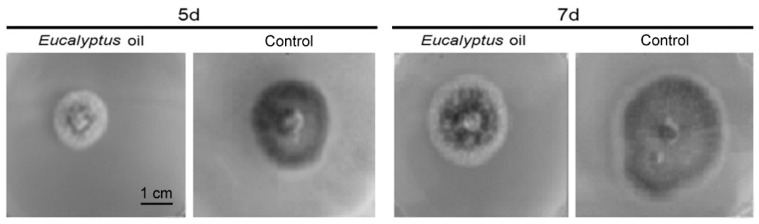
The oil impact on the growth of *M. grisea* after being cultured for 5 days and 7 days.

The oil impacts on the growth of *M. grisea* after 5 days and 7 days of culture are shown in Figure 1. Results indicate that the colony morphology of the strain was larger, the hyphae were relatively dense and more melanin was produced in the control group than in the EO-treated groups. This suggests that EO is able to inhibit hyphal growth and spore sprouting of *M. grisea*. The EO reached the highest inhibition rate to *M. grisea* after being cultured for 5 days. After 7 days of culture, the colony morphology of the strain in the EO-treated group became larger, and inhibition rate declined, and spores and melanin were produced, which may be because the oil had evaporated.

### 2.2. SEM Results

**Figure 2:**
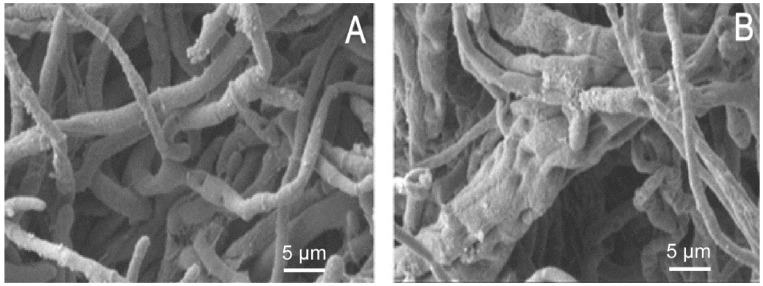
*Eucalyptus* oil effects on *M. grisea* hypha growth by the scanning electron microscopy. (**A**) control group; (**B**) experimental group.

SEM was used to examine the morphological variations of *M*. *grisea* (Figure 2). We found remarkable morphological and structural alterations of the hypha. These changes include irregular shape, floccules on the mineral surface, cavities in the outer surface, adhesion of mycelia, and swellings at the top of spores. Both the infective ability and biological activity of *M*. *grisea* were largely inhibited by *Eucalyptus* oil.

### 2.3. Hybridization Process Results

**Figure 3:**
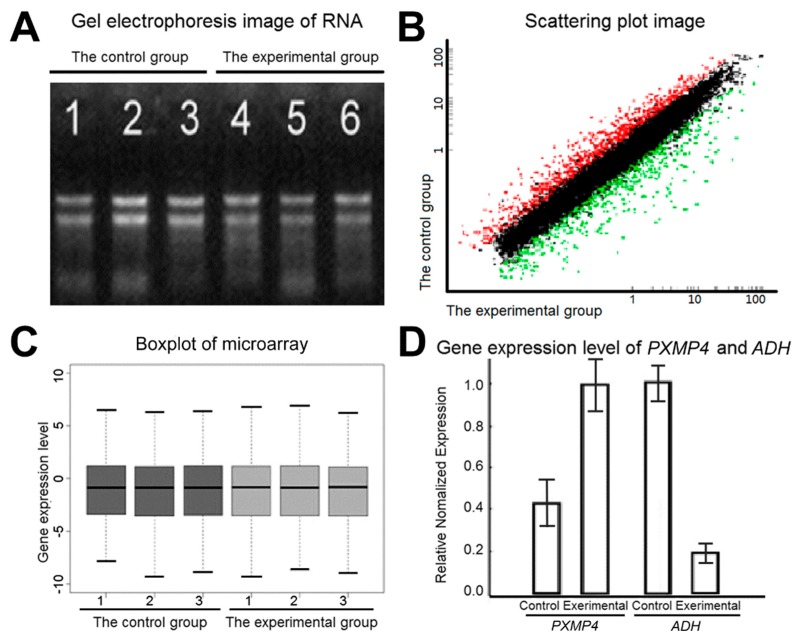
The tests for the hybridization process. RNA integrity (**A**) was assessed by standard denaturing agarose gel electrophoresis. Scatter plots (**B**) shows the correlation of signal values between control group and experimental group. The box diagram (**C**) shows the results of data processing and normalization (to the actin 1 gene). RT-PCR (**D**) shows a similar expression pattern to the corresponding results of gene chip. The up-regulated gene is peroxisomal membrane protein 4 (*PXMP4*); the down-regulated gene is alcohol dehydrogenase (*ADH*). Bars indicate SD.

RNA integrity was assessed by standard denaturing agarose gel electrophoresis (Figure 3A). The purity of RNA was A260/280 ≥ 1.80, RNA acquisition ≥1 µg. The electrophoresis of RNA on gel containing formaldehyde showed that the electrophoresis result was satisfactory with clear straps in the correct place (28S:18S rRNA) and the brightness of bands is close to or larger than 2:1. This suggests that the quality control of RNA meet the requirements. Scatter plots (Figure 3B) suggest that there was a significant relationship between the control group and the experimental group. The box diagram (Figure 3C) shows the data processing and normalization results. The mean values of all genes in the control group and the experimental group were at the same level, which reflects the differentially expressed genes between the two groups, and implies that the data were reliable. For each gene, the RT-PCR showed a similar expression pattern to the corresponding results of the gene chip (Figure 3D). The expression levels of *PXMP4* (encoding peroxisomal membrane protein 4) increased more than 2.34 times while the chips increased more than 2.24 times. The gene expression level of alcohol dehydrogenase gene *ADH* decreased 5.21 times, while that of the chips decreased 5.86 times. This shows that the results of RT-PCR were almost the same as those of the chips. In conclusion, the test results of the hybridization process show that hybridization meets the experimental requirements.

### 2.4. Microarray Data Analysis

#### 2.4.1. Identification of Differentially Expressed Genes

The *M*. *grisea* mRNA Genome Array was used for detecting the differentially expressed genes between the test strains treated by the *Eucalyptus* oil and the untreated strains. The results showed 1919 genes were significantly affected, of which 1109 were down-regulated and 810 were up-regulated (*p* < 0.05, FC > 2). 

#### 2.4.2. GO Analysis

Table 2 shows the result of functional annotation and cluster analyses. Based on biological processes, this EO treatment affects metabolism of cells, mycelium development, transmembrane transport and interaction with host via proteins. Based on molecular function, this treatment affects enzyme activities, catalytic activity and transcription factor activity. Based on the cellular component, this treatment affects integrity of membrane, cytoplasm and nucleus.

### 2.5. Analysis of Treatment Results of the Eucalyptus Oil Combined with Antifungal Drug Targets

#### 2.5.1. Inhibition to Cell-Wall Synthesis, Chitin and Cellulose Synthesis

The fungal cell wall is a special structure of fungi. Cell wall-acting antifungals are inherently selective and fungicidal, features that make them particularly attractive for clinical development. The coordinated synthesis and modification of chitin, a key component of the fungal cell wall, is essential for plant fungal pathogens to maintain cell structure integrity and full pathogenicity. Key enzymes that are related to cell-wall synthesis of fungi are ideal targets for antifungals [24]. For example, polyoxin can interfere with the synthesis of chitin, which inhibits cell-wall synthesis and results in cell death. After polyoxin contact, germ tubes and hyphae become swollen and ruptured, cell contents leak and the organism dies. Polyoxin also inhibits expansion of the disease spot and spore bearing. After the oil treatment, the expression level of chitin deacetylase gene (A_98_P124148, CDAs) decreased significantly (FC = 19.21), which assists in control of the decomposition rate and products [25,26]. Therefore, the down-regulation after treatment may influence the regulation of chitin decomposition, causing chitin to be digested, cell wall structures to be destroyed, and the formation of irregularly shaped hyphae, thus reducing the infection capacity of the strain. 

Cellulose is the main component of fungal cell walls. Antifungal agents such as propionamide can affect hyphal development and spore-bearing by influencing cellulose synthesis. Cellobiose dehydrogenase is associated with cellulose production. After the oil treatment, the expression level of cellobiose dehydrogenase gene (A_98_P175762) was significantly up-regulated (FC = 2.92). According to the SEM observations, the *Eucalyptus* oil may affect cellulose synthesis of *M. grisea*, leading to abnormal hypha growth.

#### 2.5.2. Interference with Respiration

Mitochondria are important subcellular organs that generate energy for cells. Recent studies suggested that mitochondria are related to cellular aging and cell death, and played a notable role in intracellular communication [27,28]. The antibacterial ability of α-pinene is related to its suppression of the aerobic metabolism of mitochondria [21]. Azoxystrobin can affect energy metabolism by inhibiting cellular respiration, which inhibits the hyphal growth and spore sprouting, causing eventual cell death [29]. Choline dehydrogenase is an important component of electron transport chain in the mitochondrial inner membrane. After the oil treatment, the expression level of choline dehydrogenase gene (A_98_P134755) was decreased significantly (FC = 3.94). Therefore, the down regulation after treatment may affect the respiration of *M. grisea*, leading to an unfavorable influence on energy metabolism.

Glycolytic reactions provide energy for organisms and are crucial to their function. The glycerol, which enters glycolysis, is closely related to the infection of *M. grisea*. The antimicrobial mechanism of early copper-based and mercury-based pesticides was to block glycolysis and energy metabolism by interfering with the important glycolytic enzymes, such as pyruvate kinase, hexokinase and pyruvate decarboxylase [30]. After the EO treatment, the differential expressions of genes that play a vital role in glycolysis and energy metabolism indicate that this EO treatment affects the glycolysis of *M. grisea.* Spore germination and appressorium formation of *M. grisea* require a constant source of energy, most of which is derived from oxidation of glucose. Glucose oxidase may affect the glucose metabolism and cause a deficiency of energy supply, which influences spore germination and appressorium formation of *M. grisea*, and its physiological activity and pathogenicity [31]. After the oil treatment, the expression level of glucose oxidase gene (A_98_P114990) decreased significantly (FC = 3.50). Phosphoglycerate kinase (PGK) as a key glycolytic enzyme, is necessary for life and the lack of PGK results in severe dysfunction, such as a metabolic disorder [32]. After the oil treatment, the expression level of *PGK* (A_98_P132671) decreased significantly (FC = 4.47). Therefore, the down regulation after treatment may affect the glycometabolism and energy metabolism of *M. grisea*, leading to abnormal hyphae growth and weakening of the pathogenicity of hyphae and spores.

#### 2.5.3. Inhibition of Nucleic Acid Synthesis

After the oil treatment, the expression level of ribose 5-phosphate isomerase gene (A_98_P136903) was decreased significantly (FC = 2.01). Nucleotide coenzymes and nucleic acid were synthesized with 5-phosphate isomerase as the main materials, which are well known for healing effects after injury (tissue regeneration) [33]. Therefore, the down regulation after treatment may also affect self-healing and tissue regeneration.

### 2.6. Potential Application

*Eucalyptus* oil can be regarded as a new original plant biopesticide that is highly efficient, broad spectrum and safe. The practical application of *Eucalyptus* oil as a biological pesticide should involve consideration of the application modes and time according to the different bacteriostatic rates (*i.e.*, weather, temperature, and other properties). Besides, some additives to the *Eucalyptus* oil also should be developed to reduce its volatilization.

According to GO annotation analysis, this EO treatment affect mycelium development, nitrogen metabolism, carbohydrate metabolism, lipid metabolism, protein synthesis, protein synthesis, signal transduction and material transport in *M. grisea.* This may cause abnormal structures and physiological function disorder in *M. grisea* and correspondingly reduce its survival and infective ability. The SEM result showed that the *Eucalyptus* oil plays an important role in damaging mycelium morphology and structure. The influence of this EO treatment on the growth of *M. grisea* conforms to some current antifungal drug targets, and thus has the potential to be developed into a future biological pesticide.

The *Eucalyptus* oil resulted in at least 80% control of rice blast when applied at concentrations over 2.5 mg/mL. Minimum inhibitory concentrations(MIC) values between 0.55 and 4.45 mg/mL (geometric mean 1.26 mg/mL) were found against 22 different *Malassezia furfur* strains while very low MIC values for miconazole were found for *M. furfur* (geometric mean 2.34 mg/mL) [34]. 

## 3. Experimental Section

### 3.1. Extraction of Eucalyptus Oil (EO)

The leaves (3 years of growth) of *E. grandis* × *E. urophylla* were collected from Yuping, Hongya, a small town in Sichuan Province, China, in August 2012. The fresh leaves were harvested at random from 15–20 trees in a forest. After natural drying, grinding and sieving (20 mesh) of leaf samples, 100 g of samples were collected. Oils were extracted from the leaves of *Eucalyptus* using a laboratory scale supercritical fluid extraction (SFE) system using CO_2_. The oil extracted by hydro-distillation contained only volatile compounds while the oil from the SFE and Soxhlet contained both volatile and higher molecular weight compounds [35].

The oil was extracted as follows: extraction temperature of 80 °C, extraction time of 8 h, and an extraction pressure of 400 MPa. The resultant yield of oil was 7.87%. The EO was preserved at 4 °C in a dark brown vial under shaded conditions until further analysis [16]. 

### 3.2. Inhibitory Test

Fungal strains including *Fusarium graminearum*, *Rhizoctonia solani*, *Magnaporthe grisea (A47)*, *Colletotrichum gloeosporioides*, *Alternuria longipes*, *Alternaria solani*, *Botrytis cinerea*, *Bipolaris maydis*, *Fusarium moniliforme*, *Rhizoctonia solani* and *Setosphaeria turcica* were obtained from the Institute of Plant Protection, Sichuan Academy of Agricultural Sciences (Chengdu, China). Fungal strains were maintained on potato dextrose agar (PDA) medium. Fungal cultures were sub-cultured (1% inoculum) in PDA broth at 28 °C for at least 2–4 days before their use in the screening assays.

Oil dilutions were prepared by adding 500 μL (0.396 g) of oil to Tween 20 with final concentration of 1% (*w*/*v*), then we added distilled water to dilute samples to the treatment concentrations. An antifungal activity test was carried out using the mycelium growth rate method at 28 °C for 5 days [36]. The oil was mixed with the melting PDA medium, until a final test concentration of 2.5 mg/mL was reached. Tween 20 was used as cosolvent. Blank controls were the fungi cultured in normal PDA medium and negative controls were the fungi cultured in PDA medium with Tween 20.

### 3.3. Morphological Study of the Inhibitory Activities of EO on M. grisea

The samples were prepared as described in the Section 3.2. Scanning electron microscopy (SEM, Gemini, Carl-Zeiss Comp., Schwabhausen, Germany) was used to examine the morphological variations of *M*. *grisea* after the 5-days treatment described previously.

### 3.4. Total RNA Extraction

*M*. *grisea* was cultured as previously described, leading to final test concentrations of EO at 2 mg/mL. The total RNA was extracted using Trizol (Invitrogen, Gaithersburg, MD, USA) and purified with mirVana miRNA Isolation Kit (Ambion, Austin, TX, USA) according to manufacturer’s protocols. RNA integrity was determined by capillary electrophoresis using the RNA 6000 Nano Lab-on-a-Chip kit and the Bioanalyzer 2100 (Agilent Technologies, Santa Clara, CA, USA). Only RNA extracts with RNA integrity number values >6 underwent in further analysis. Furthermore, the total RNA was isolated and purified using a NucleoTrap^®^RNA clean-up Kit (Macherey-Nagel, Düren, Deutschland), and analyzed by agarose gel electrophoresis.

### 3.5. Fluorescent Dye Labelling, Chip Hybridization and Scanning

Subsequent data processing was analyzed for data summarization, normalization and quality control by using the GeneSpring GX software (Capital Bio, San Diego, CA, USA) [37]. Normalization was conducted using the percentile shift method. To screen the genes differentially expressed by threshold values of ≥2 and ≤−2-fold change and a Benjamini-Hochberg corrected *p* value of 0.05, and Flag was marked as Detected. The data was Log2 transformed and median centered by genes using of CLUSTER 3.0 software (CapitalBio, Beijing, China) [38]. The functional enrichment of up- or down-regulated genes was assessed based on the GO terms [39].

### 3.6. RT-PCR Analysis

RT-PCR was used to validate the results for two genes from *M. grisea*. Actin was used as the reference gene. Gene expression level of peroxisomal membrane protein 4 and alcohol dehydrogenase after the treatment was compared with the results of gene chip. Primer design: Actin 1-F: 5′-TCGACGTCCGAAAGGATCTGT-3′; Actin 1-R: 5′-ACTCCTGCTTCGAGATCCACATC -3′; Peroxisomal membrane protein 4-F: 5′-GCCCGTATTCGCTGCCACAA-3′; Peroxisomal membrane protein 4-R: 5′-CTCAGGCCATCCCATTCGTT-3′; Alcohol dehydrogenase-F: 5′-AGATGCCCAGCGTCATTTCC-3′; Alcohol dehydrogenase-R: 5′-GCCAATGTGCTTCTTGTTCCA-3′.

### 3.7. Statistical Analysis

All results presented here were confirmed in at least three independent experiments. Data were expressed as mean ± SD. Statistical comparisons were made by Student’s *t*-test. A value of *p* < 0.05 was considered statistically significant.

## 4. Conclusions

Expression pattern of genes that play an important part in glycolysis and energy metabolism indicated that *Eucalyptus* oil can influence the normal physiology activity of fungal pathogens, probably through inhibiting glycolysis which in turn influenced cell energy metabolism. This means that the original balance of *M. grisea* is broken. Furthermore, the *Eucalyptus* oil had characteristics of high activity and low toxicity, therefore, it could be developed as an ideal pesticide for the agricultural industry. *Eucalyptus* oil could have direct potential as a green biopesticide because of its high content of antifungal components.

## Figures and Tables

**Table 1:** Inhibitory activities of the *Eucalyptus* oil on plant pathogenic fungi.

Strain	Control Group (cm)	EO-Treated Group (cm)	Inhibition Rate (%)
*Setosphaeria turcica*	1.7 ± 0.3	0.6 ± 0.2	91 ± 9
*Magnaporthe grisea*	2.4 ± 0.4	1.5 ± 0.3	81 ± 7
*Botrytis cinerea*	3.0 ± 0.8	1.1 ± 0.3	75 ± 7
*Fusarium graminearum*	5.2 ± 1.7	1.7 ± 0.4	74 ± 6
*Bipolaris maydis*	5.9 ± 1.6	2.6 ± 0.6	62 ± 6
*Rhizoctonia solani*	5.4 ± 1.5	2.6 ± 0.8	56 ± 5
*Rhizoctonia solani*	3.5 ± 1.0	2.1 ± 0.5	46 ± 5
*Colletotrichum gloeosporioides*	2.9 ± 0.6	2.0 ± 0.4	45 ± 5
*Alternuria longipes*	4.0 ± 1.1	2.9 ± 0.7	31 ± 5
*Alternaria solani*	3.6 ± 1.0	2.8 ± 0.7	26 ± 4
*Fusarium moniliforme*	4.3 ± 1.1	3.3 ± 0.7	25 ± 4

Diameters of the plaques and inhibition rates (determined by the mycelium growth rate method) are shown.

**Table 2:** GO analysis for differentially expressed genes.

GO Terms	Terms Type	Genes_In_Term	DEG	Up	Down	*p*-Value
GO:0055114 (oxidation-reduction process)	P	777	109	51	58	1.53 × 10^−10^
GO:0044271 (cellular nitrogen compound biosynthetic process)	P	38	14	0	14	1.01 × 10^−5^
GO:0043581 (mycelium development)	P	468	59	22	37	5.60 × 10^−4^
GO:0009103 (lipopolysaccharide biosynthetic process)	P	10	6	4	2	6.24 × 10^−4^
GO:0006066 (alcohol metabolic process)	P	13	6	4	2	3.37 × 10^−3^
GO:0055085 (transmembrane transport)	P	292	37	10	27	1.06 × 10^−2^
GO:0052051 (interaction with host via protein secreted by type II secretion system)	P	50	11	9	2	1.15 × 10^−2^
GO:0008152 (metabolic process)	P	328	39	27	12	1.69 × 10^−2^
GO:0005375 (copper ion transmembrane transporter activity)	P	8	4	1	3	1.69 × 10^−2^
GO:0035434 (copper ion transmembrane transport)	P	8	4	1	3	1.69 × 10^−2^
Total P		1992	289	129	160	
GO: 0016491 (oxidoreductase activity)	F	268	50	23	27	6.79 × 10^−9^
GO:0050660 (flavin adenine dinucleotide binding)	F	90	18	11	7	2.27 × 10^−3^
GO:0008812 (choline dehydrogenase activity)	F	9	5	3	2	6.47 × 10^−3^
GO:0004181 (metallocarboxypeptidase activity)	F	6	4	1	3	9.44 × 10^−3^
GO:0003824 (catalytic activity)	F	175	24	11	13	2.44 × 10^−2^
GO:0000981 (sequence-specific DNA binding RNA polymerase II transcription) factor activity)	F	113	17	11	6	3.99 × 10^−2^
Total F		661	118	60	58	
GO:0016021 (integral to membrane)	C	742	52	21	31	3.37 × 10^−6^
GO:0005737 (cytoplasm)	C	259	20	1	19	8.13 × 10^−3^
GO:0005634 (nucleus)	C	610	35	18	17	1.41 × 10^−2^
Total C		1611	107	40	67	

DEG = differentially expressed genes; P: biological_process; C: cellular_component; F: molecular_function.
